# WHY NOT LONG-TERM CARE? EXPLORING STUDENT NURSES’ PERSPECTIVES ON CAREERS CARING FOR OLDER ADULTS

**DOI:** 10.1093/geroni/igae098.3931

**Published:** 2024-12-31

**Authors:** Kristen Childress, Angela Kokinakos

**Affiliations:** University of Washington, Seattle, Washington, United States; Phoenix Psychiatric Services, PLLC, Seattle, Washington, United States

## Abstract

The aging population and growing numbers of people living with Alzheimer’s Disease and related dementias (ADRD) are increasing the demand for licensed nurses, especially in the long-term care (LTC) sector. More than 89% of long-term care facilities in the United States (US) report ongoing staffing shortages that persist despite recruitment and retention efforts, directly impacting quality of care and outcomes for people with ADRD. Existing evidence describes factors influencing nursing staff retention in LTC but there is a paucity of information related to factors that impact nursing students’ decision-making related to entering careers in LTC. The purpose of this qualitative study was to understand student nurses’ perceptions of LTC as a career path. The first of four focus groups was conducted with five individuals. Interviews revealed that most participants plan to work in the hospital following graduation. Themes related to lack of plans to work in LTC included limitations in career advancement, past experiences with LTC culture, and perceptions of the care provided in LTC. Pay was a mixed factor, with quality of the work environment being more important to participants. Participants felt that faculty representation of LTC careers was positive, but participants described limited exposure to gerontologic nursing and LTC as part of their training, indicating that modification of these factors might have changed their perspective of careers in LTC. These early results identify key areas where crafting and delivering positive messaging and implementing educational interventions could boost nursing students’ interest in LTC careers.

